# Comparison of human lung tissue mass measurements from Ex Vivo lungs and high resolution CT software analysis

**DOI:** 10.1186/1471-2466-12-18

**Published:** 2012-05-14

**Authors:** Erik Henne, Joseph C Anderson, Norma Lowe, Steven Kesten

**Affiliations:** 1 Uptake Medical®, 1173 Warner Ave, Tustin, CA, 92780, USA

**Keywords:** Emphysema, Software, Computed tomography, Volume, Mass, Measurement lung volume reduction

## Abstract

**Background:**

Quantification of lung tissue via analysis of computed tomography (CT) scans is increasingly common for monitoring disease progression and for planning of therapeutic interventions. The current study evaluates the quantification of human lung tissue mass by software analysis of a CT to physical tissue mass measurements.

**Methods:**

Twenty-two ex vivo lungs were scanned by CT and analyzed by commercially available software. The lungs were then dissected into lobes and sublobar segments and weighed. Because sublobar boundaries are not visually apparent, a novel technique of defining sublobar segments in ex vivo tissue was developed. The tissue masses were then compared to measurements by the software analysis.

**Results:**

Both emphysematous (n = 14) and non-emphysematous (n = 8) bilateral lungs were evaluated. Masses (Mean ± SD) as measured by dissection were 651 ± 171 g for en bloc lungs, 126 ± 60 g for lobar segments, and 46 ± 23 g for sublobar segments. Masses as measured by software analysis were 598 ± 159 g for en bloc lungs, 120 ± 58 g for lobar segments, and 45 ± 23 g for sublobar segments. Correlations between measurement methods was above 0.9 for each segmentation level. The Bland-Altman analysis found limits of agreement at the lung, lobe and sublobar levels to be −13.11% to −4.22%, –13.59% to 4.24%, and –45.85% to 44.56%.

**Conclusion:**

The degree of concordance between the software mass quantification to physical mass measurements provides substantial evidence that the software method represents an appropriate non-invasive means to determine lung tissue mass.

## Background

Quantification of lung tissue via analysis of computed tomography (CT) scans is increasingly common to understand disease processes [1,2]. Recently, quantitative analysis has been utilized for planning and follow-up of therapeutic interventions, such as bronchoscopic thermal vapor ablation (BTVA^TM^) for lung volume reduction in patients with emphysema. BTVA delivers a specific amount of thermal energy based on the tissue mass of each lung segment customized for each person (i.e. calories per gram of tissue) [3].

Automatic segmentation and measurement of tissue mass of the lung, lobes, and sublobar segments from CT scans can be computed. Three main algorithmic approaches have been reported in the literature. 1) Approaches based on the explicit segmentation of fissures [4]. 2) Approaches using supervised classification schema [5], which are particularly sensitive to fissure incompleteness, nearby tumors, atelectasis or emphysematous changes. 3) Approaches combining multiple sources of information such as bronchi segmentation, vessel segmentation, and absence of larger vessels in proximity to the lobar boundaries to define robust 3D sublobar segments [6]. The lobar algorithm studied in this paper falls in this third category and is, to our knowledge, the only such software commercially available (Pulmonary Workstation 2.0, VIDA Diagnostics, Iowa City, IA). It relies on a validated airway segmentation algorithm [7-9] and a distinctive processing of the fissure surface at the interface between lobes. Tshcirren et al recommended additional quantitative analysis with in vivo data; however, to date no tissue mass or volume quantification comparative data has been published for lung, lobar, and sublobar dissection.

The current study evaluated the software’s tissue mass quantification function using human lung tissue. Segmentation of ex vivo lungs at the lobar level was identified by the visible fissures between lobes. However, segmentation at the sublobar level is more difficult because sublobar boundaries are not visually apparent. A novel technique of defining sublobar segments in ex vivo lung tissue was developed for this study using an occlusion balloon to preferentially inflate sublobar segments. This study assessed the agreement of the software tissue mass quantification at multiple anatomical lung levels using tissue from the aforementioned technique of lung, lobar, and sublobar segmentation.

## Methods

### Human lung acquisition and preparation

Non-transplantable excised en bloc human lungs were obtained from two tissue procurement agencies (International Institute for the Advancement of Medicine, Pennsylvania, USA and National Resource Center, Pennsylvania, USA). The tissue requests were approved by an external feasibility committee for each agency. No conditions were placed on age, sex, or patient height variables as the lungs were accepted from the organizations. Both non-emphysematous and emphysematous lungs were included in the study. Emphysematous lungs were visually identified by the presence of hyperinflation, bullae, scarring, and deformities. Lungs were excluded from the study if they had significant fluid accumulation or did not inflate properly. During lung preparation, non-lung tissue, such as fat and blood vessels, was removed from the lungs.

### Imaging

**Figure 1:**
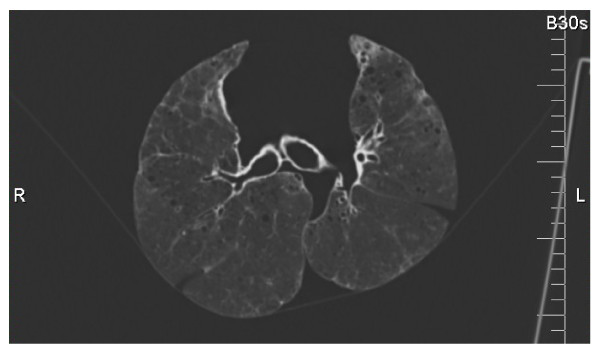
A sample CT image of an ex vivo human lung cradled in the specially designed hammock.

The lungs were placed in a non-metallic hammock specially designed to cradle the lungs in a supine position with minimal deformation (Figure 1). The lungs were inflated with static pressure within the range of 20–30 cm H_2_O to simulate total lung capacity. The entire lungs were scanned by CT according to the software specifications, slice thickness of ≤ 1 mm, slice separation of 0.5 mm, exposure 40–200 mAs, tube voltage 120 kVP, Siemens B30 kernel.

A blinded copy of the CT scan was sent to the software developer (VIDA Diagnostics, Iowa City, Iowa) for analysis after tissue dissection occurred. Blood and chest wall are two of the landmarks used by the software that are missing in ex vivo tissue. Therefore additional editing of the analysis by the software technician was required to help define the lung boundary and distinguish exsanguinated blood vessels from airways. The quantitative analysis report from the software provided segment labeling, airway diameter, total volume, air only volume, and tissue only volume. These values were provided for the lung, lobar, and sublobar segment level. Tissue only volume was converted to tissue mass by multiplying by the density 1.0 g/ml [10].

### Human lung dissection

**Figure 2:**
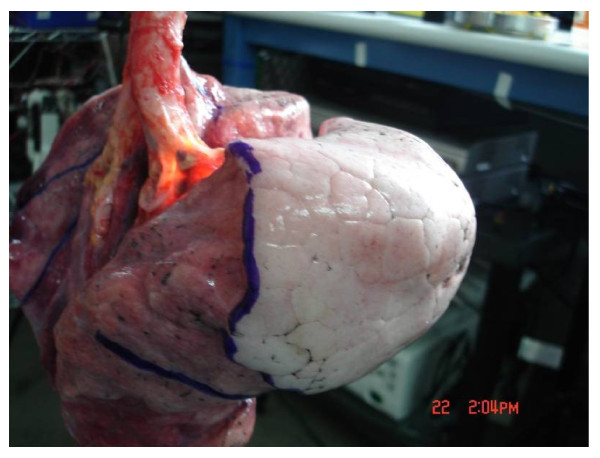
**A lung is suspended from the stand and a catheter with compliant balloon is used to inflate the right upper lobe apical segment (RB1).** The boundary between the inflated segment and the non-inflated segments is demarcated.

The deflated lungs were suspended from a stand and a bronchoscope was used to navigate to one of the upper sublobar segments. A catheter with a compliant balloon at the tip was used to occlude a segment. A nitrogen source was attached to the catheter and the segment was inflated with 10 to 30 cm of H_2_O until a clear demarcation between the target segment and the other segments was visually apparent on the lung surface. Inadequate pressure could cause the segment to not inflate completely. The segment was considered optimally inflated when the demarcation was visually most apparent. A surgical marker was used to trace the visible demarcation on all sides of the lobe (Figure 2). The segment was inflated gradually to preclude inflation of neighboring segments through higher resistance collateral ventilation, particularly prevalent in diseased tissue. Irreversible inflation of neighboring segments through collateral ventilation occurred approximately five times. In these instances the lung was placed in a container at approximately 40° F and after two to four hours the trapped gas dissipated and the procedure was repeated. If adequate boundary demarcation could not be achieved, the segment was not included in the final analysis (5% excluded).

Only the upper lobes were divided into sublobar segments. The right upper lobe was divided into the apical (RB1), posterior (RB2), and anterior (RB3) sublobar segments. The left upper lobe was divided into the apicoposterior (LB1 & LB2, or LB1 + 2) and the anterior (LB3). When apicoposterior/anterior segment demarcation was not possible, which occurred in 30% of left lungs, the upper lobe was divided into the superior division (LB123) and the lingula (LB45).

**Figure 3:**
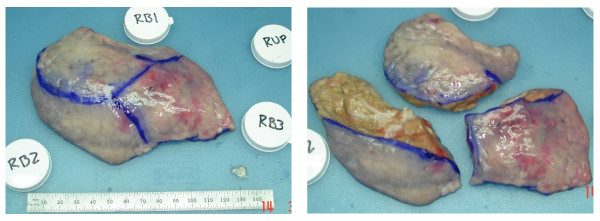
**Right upper lobe separated from other lobes after demarcation (left).** Sublobar segments separated by cutting from surface mark to airway trifurcation (right).

After demarcation was completed, the en bloc lung was weighed. All of the lobes were then separated along fissures and the lobes were weighed. For each upper lobe, sublobar segments were separated by cutting through the tissue along a plane between the surface demarcation lines and the segment airway bifurcation/trifurcation (Figure 3). The separated sublobar segments were then also weighed. The person performing the dissection was blinded to the software quantitative analysis.

### Statistical analysis

Values from the dissection weighing and the software measurement were compared using the Pearson product–moment correlation coefficient and the method described by [Bland and Altman 11,^12^]. Because the average absolute measurement difference increases with tissue size, the relative agreement ([software – dissection]/dissection) was calculated and used with the Bland Altman method.

## Results

### Lung segments assessed

The study included exsanguinated en-bloc lungs from twenty-two patients. Donor and tissue characteristics are shown in Table 1. Approximately eight additional lungs could not be included because of procurement effects, such as lacerations, or noticeable consolidation that would affect weight measurement results. Both emphysematous (n = 14) and non-emphysematous (n = 8) lungs were included in the study. Each lung was dissected into the five lobes to yield 110 lobes. Each upper lobe was demarcated and dissected as described. If an adequate demarcation could not be obtained and dissection was not possible, the segment was excluded from the study. The dissection of the right upper lobes yielded 62 sublobar segments (6% excluded) and the dissection of the left upper lobes yielded 67 sublobar segments (4% excluded).

**Table 1:** Baseline characteristics of tissue in the study

**Parameter**	**Value**
Number of lungs	22
Mean donor age (range)	49 (18 to 65) years
Mean donor height (range)	173 (152 to 191) cm
Donor sex (male : female)	10 : 12
Disease state (non-emphysematous : emphysema)	8 : 14
Number of lobes	110
Number of right upper sublobar segments (RB1, RB2, RB3)	62 (22, 20, 20)
Number of left upper sublobar segments (LB1, LB2, LB3, LB1 + 2, LB123, LB45)	67 (7, 2, 12, 7, 19, 20)

### Tissue mass

**Figure 4:**
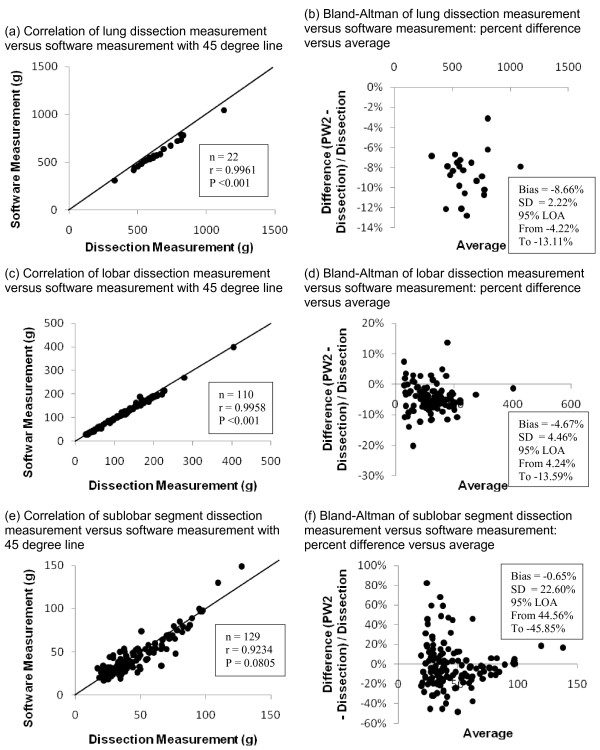
**Correlation and Bland-Altman plots comparing dissection and software methods: (a) Correlation plot comparing bilateral lung mass measured by dissection and software methods.(b)** Bland-Altman plot: agreement between paired measurements of bilateral lung mass measured by dissection and software methods. **(c)** Correlation plot comparing lobar segment mass measured by dissection and software methods. **(d)** Bland-Altman plot: agreement between paired measurements of lobar segment mass measured by dissection and software methods. **(e)** Correlation plot comparing sublobar segment mass measured by dissection and software methods. **(f)** Bland-Altman plot: agreement between paired measurements of sublobar segment mass measured by dissection and software methods.

Masses (Mean ± SD) as measured by dissection were 651 ± 171 g for en bloc lungs, 126 ± 60 g for lobar segments, and 46 ± 23 g for sublobar segments. Masses as measured by software analysis were 598 ± 159 g for en bloc lungs, 120 ± 58 g for lobar segments, and 45 ± 23 g for sublobar segments. Results are summarized in Table 2. Correlations between measurement methods (Figure 4a, c, e) were above 0.9 for each segment level.

**Table 2:** Results of mass measurements for the entire lung, lobar segments and sublobar segments

**Parameter**	**Dissection measurement (mean ± SD)**	**Software measurement (mean ± SD)**
Bilateral lung	651 ± 171 g	598 ± 159 g
Lobar segments	126 ± 60 g	120 ± 58 g
Sublobar segments	46 ± 23 g	45 ± 23 g

The difference between the dissection method measurement and the software measurement was calculated for each lung, lobe, and sublobar segment and divided by the dissection measurement. This relative difference was used because there is a relationship between segment size and absolute measurement agreement. These relative differences were plotted verses the average for each measurement to generate a Bland-Altman Plot (Figure 4b, d, f). The average (μ) and standard deviation (σ) of the relative measurement differences was calculated. The limits of agreement (LOA) were calculated (μ ± 2σ) for each mass measurement (Table 2). The LOA at the lung and lobar level were in high concordance, with an interval of 9% and 18% respectively. The LOA interval at the sublobar level was 90%. A small but consistent bias (p < 0.001) was found for lung (9%) and lobar (5%) measurements (dissection measurements were larger). No consistent bias was found at the sublobar level (p = 0.081). These results are summarized in Table 3.

**Table 3:** Summary of Bland-Altman analyses of correlation of mass measurements determined from dissection and software analyses methods

		**Correlation coefficient**		**95% limits of agreement (%)**	
**Measurement**	**Number**		**Bias (%)**	**From**	**To**
Bilateral lung	22	0.9961	−8.66%	−13.11%	−4.22%
Lobar segments	110	0.9958	−4.67%	−13.59%	+4.24%
Sublobar segments	129	0.9234	−0.65%	−45.85%	+44.56%

## Discussion

Lung tissue quantification is useful for monitoring the progression of many diseases [1] and with the advent of novel interventional therapies [3], non-invasive lung quantification (e.g. volume, mass, airway diameter) is increasingly important. We were motivated to further develop techniques for lung segmental tissue mass assessment and validation of commercially available software algorithms because of our interest in bronchoscopic lung volume reduction through the application of thermal energy (i.e. bronchoscopic thermal vapor ablation or BTVA). The aim of this study was to evaluate the software CT analysis of tissue mass using actual ex vivo human lung tissue mass measurements.

To evaluate the agreement of the CT analysis measurement, excised en-bloc lungs were scanned with CT and analyzed by software. The same lungs were then dissected and the segments weighed. Because there are no visible sublobar divisions in the human lung, an innovative technique was developed to selectively inflate sublobar segments to enable demarcation. Measurements were compared at the bilateral lung, lobar, and sublobar level (upper lobes only). Bland-Altman analysis was used to determine the bias and limits of agreement (LOA) for each measurement comparison.

The correlation and LOA between the dissection and software method indicated substantial agreement. The correlation was closest (r > 0.99) at the bilateral lung and lobar level. A small bias was observed, which is most likely due to extraneous tissue, such as bronchi, that the software excluded but was included when the lung was weighed as a whole. When the lobar and sublobar dissections were performed extraneous tissue was removed which reduced the bias at those levels.

The sublobar comparison showed no technique bias; however, the sublobar LOA were larger relative to the lobar measurements. The LOA were expected to increase with sublobar samples because the samples were physically smaller and therefore the precision was reduced. Additionally, some of the increase in the LOA is likely due to the decreased feasibility of precise dissection at the sublobar level as compared to the lobar level. Because the same tissue was used for lobar and sublobar analysis, the increase in LOA is most likely related to segmentation of the sublobar tissue rather than quantification of the tissue. However, the attribution of the decrease in agreement from the dissection method versus the software algorithm is unknown. Nevertheless, given the inherent issues of dissection, the degree of concordance provides substantial evidence that the software method represents an appropriate non-invasive means to determine tissue mass.

The data presented is relevant to current and future developments in the treatment of lung disease. Interventional bronchoscopy is a developing field in which significant advances have recently occurred in the treatment of advanced emphysema. Lung volume reduction can be achieved through the bronchoscopic application of valves, coils, sealants, and thermal energy in the form of water vapor. However, these methods require a thorough analysis and understanding on lung anatomy and tissue [12-15]. The analysis method must be non-invasive and provide sufficiently accurate information in order to apply these new technologies optimally. In particular, BTVA utilizes a personalized approach to treatment where each vapor application is individually determined based on the patient’s sublobar lung tissue mass. Hence, the results of non-invasive tissue assessment (i.e. quantitative CT analysis) are important in optimizing therapeutic treatment.

## Conclusion

While other aspects of computerized quantification of lung tissue have been validated, no comparison of the tissue mass quantification has been published to date with human lung at the sublobar level. This study describes a novel method of demarcating sublobar segments. The method can be used to dissect and weigh ex vivo tissue and use the results to evaluate the measurements of a CT quantification method. The data demonstrate a high level of agreement between a software mass quantification and ex vivo tissue dissection. This analysis provides confirmatory data regarding the interpretation of software quantification of lung tissue mass for current and future clinical applications to the treatment of lung disease.

## Abbreviations

BTVA: Bronchoscopic thermal vapor ablation; CT: Computed tomography; HRCT: High resolution computed tomography; LB1: Left upper apicoposterior sublobar segment; LB123: Left upper superior sublobar segment; LB1 + 2: Left upper apicoposterior sublobar segment; LB2: Left upper apicoposterior sublobar segment; LB3: Left upper anterior sublobar segment; LB45: Left upper lingula sublobar segment; LOA: Limits of agreement; RB1: Right upper apical sublobar segment; RB2: Right upper posterior sublobar segment; RB3: Right upper anterior sublobar segment; SD: Standard deviation.

## Competing interests

All authors are employees of Uptake Medical®.

## Authors’ contributions

EH: Contributed to study design, statistical analysis, and manuscript preparation. Developed dissection method, performed dissection, measurements, and data management. JCA: Contributed to study design, statistical analysis, and manuscript preparation. NL: Contributed to study design and manuscript preparation. SK: Contributed to manuscript preparation. All authors read and approved the final manuscript.

## Pre-publication history

The pre-publication history for this paper can be accessed here:

http://www.biomedcentral.com/1471-2466/12/18/prepub
